# Carbapenem-Resistant *Acinetobacter baumannii* in Three Tertiary Care Hospitals in Mexico: Virulence Profiles, Innate Immune Response and Clonal Dissemination

**DOI:** 10.3389/fmicb.2019.02116

**Published:** 2019-09-20

**Authors:** María Dolores Alcántar-Curiel, Roberto Rosales-Reyes, Ma Dolores Jarillo-Quijada, Catalina Gayosso-Vázquez, José Luis Fernández-Vázquez, José Eduardo Toledano-Tableros, Silvia Giono-Cerezo, Paola Garza-Villafuerte, Arath López-Huerta, Daniela Vences-Vences, Rayo Morfín-Otero, Eduardo Rodríguez-Noriega, María del Rocío López-Álvarez, María del Carmen Espinosa-Sotero, José Ignacio Santos-Preciado

**Affiliations:** ^1^Laboratorio de Infectología, Microbiología e Inmunología Clínicas, Unidad de Investigación en Medicina Experimental, Facultad de Medicina, Universidad Nacional Autónoma de México, Mexico City, Mexico; ^2^Departamento de Microbiología, Escuela Nacional de Ciencias Biológicas, Instituto Politécnico Nacional, Mexico City, Mexico; ^3^Hospital Civil de Guadalajara Fray Antonio Alcalde, Instituto de Patología Infecciosa y Experimental, UDG, Guadalajara, Mexico; ^4^Hospital Regional General Ignacio Zaragoza, ISSSTE, Mexico City, Mexico; ^5^Unidad de Pediatría, Hospital General de México Eduardo Liceaga, Mexico City, Mexico

**Keywords:** *Acinetobacter baumannii*, multidrug resistance, MLST, clonal dissemination, biofilm, adherence/invasion, immune response, Mexico

## Abstract

*Acinetobacter baumannii* is one of the most important nosocomial pathogens distributed worldwide. Due to its multidrug-resistance and the propensity for the epidemic spread, the World Health Organization includes this bacterium as a priority health issue for development of new antibiotics. The aims of this study were to investigate the antimicrobial resistance profile, the clonal relatedness, the virulence profiles, the innate host immune response and the clonal dissemination of *A. baumannii* in Hospital Civil de Guadalajara (HCG), Hospital Regional General Ignacio Zaragoza (HRGIZ) and Pediatric ward of the Hospital General de México Eduardo Liceaga (HGM-P). A total of 252 *A. baumannii* clinical isolates were collected from patients with nosocomial infections in these hospitals between 2015 and 2016. These isolates showed a multidrug-resistant profile and most of them only susceptible to colistin. Furthermore, 83.3 and 36.9% of the isolates carried the *bla*_OXA–__24_ and *bla*_TEM–__1_ genes for resistance to carbapenems and β-lactam antibiotics, respectively. The clonal relatedness assessed by pulsed-field gel electrophoresis (PFGE) and by multi-locus sequence typing (MLST) demonstrated a genetic diversity. Remarkably, the ST136, ST208 and ST369 that belonged to the clonal complex CC92 and ST758 and ST1054 to the CC636 clonal complex were identified. The ST136 was a high-risk persistent clone involved in an outbreak at HCG and ST369 were related to the first carbapenem-resistant *A. baumannii* outbreak in HRGIZ. Up to 58% isolates were able to attach to A549 epithelial cells and 14.5% of them induced >50% of cytotoxicity. A549 cells infected with *A. baumannii* produced TNFα, IL-6 and IL-1β and the oxygen and nitrogen reactive species that contributes to the development of an inflammatory immune response. Up to 91.3% of clinical isolates were resistant to normal human serum activity. Finally, 98.5% of the clinical isolates were able to form biofilm over polystyrene tubes. In summary, these results demonstrate the increasingly dissemination of multidrug-resistant *A. baumannii* clones in three hospitals in Mexico carrying diverse bacterial virulence factors that could contribute to establishment of the innate immune response associated to the fatality risks in seriously ill patients.

## Introduction

The World Health Organization (WHO) has listed antibiotic resistance as one of the most important problem in human health worldwide (World Health Organization [WHO], 2014). Members of the ESKAPE group are bacteria that rapidly increase their antimicrobial resistance and present new paradigms in the pathogenesis, transmission and resistance of infectious diseases (Santajit and Indrawattana, 2016). Within the most critical category of this group, *Acinetobacter baumannii* is considered as a serious threat in hospitals, particularly when its infects critically ill individuals (World Health Organization [WHO], 2017).

*Acinetobacter baumannii* has emerged as the most important nosocomial pathogen causing lethal infections in intensive care units (Ambrosi et al., 2017). The eradication of *A. baumannii* from different environments in hospital fails in part due to the ability of this microorganism to persist on diverse surfaces (Cerqueira and Peleg, 2011). *A. baumannii* is frequently involved in ventilator-associated pneumonia, wound infections, urinary tract infections, bacteremia and meningitis (Cerqueira and Peleg, 2011). This bacterium is frequently associated with nosocomial outbreaks in hospitals with high rates of mortality (Almasaudi, 2018). In recent years, the antibiotic resistance has increased rapidly and the availability of molecular epidemiology techniques have been use to establish new strategies to control the spread of carbapenem-resistant *A. baumannii* (Cieslinski et al., 2013).

*Acinetobacter baumannii* has high levels of intrinsic resistance to a number of antibiotics and a predisposition to acquire antibiotic resistance genes has contributed to increase its antimicrobial resistance patterns (Runnegar et al., 2010), generating thus new pathogens that are more complex to treat, and pose important challenges for the clinician (Fishbain and Peleg, 2010). Carbapenems are antibiotics used to treat *A. baumannii* infections (Fonseca et al., 2013) and their increased resistance to carbapenems is due to the production of carbapenemases (class D enzymes or oxacillinases and class B enzymes or metallo-β-lactamase) overexpression of efflux pumps, genetic alterations of penicillin binding proteins (PBP) and loss of porins expression (Rosales-Reyes et al., 2017).

In recent years, several virulence factors have been identified in *A. baumannii* (Harding et al., 2018) including: (a) lipopolysaccharide (LPS) which is one of the most important factors because it is involved in the innate immune responses (Erridge et al., 2007), (b) the outer membrane protein OmpA which is involved in biofilm formation on abiotic surfaces and in epithelial cell invasion (Choi et al., 2008a; Gaddy et al., 2009), (c) the metal acquisition system associated with persistence within epithelial cells, which can cause cell damage and animal death (Gaddy et al., 2012), (d) the bacterial Type VI Secretion System involved in bacterial competence (Carruthers et al., 2013; Weber et al., 2013), associated with persistence and development of bacteremia (Repizo, 2017), and (e) the resistance of some clones to complement-mediated normal human serum activity (Rosales-Reyes et al., 2017).

Although recent genomic and phenotypic analyses of *A. baumannii* have been focused in virulence factors involved in pathogenicity, we believe it’s necessary to determine its role associated to bacterial survival and persistence in the environment of the hospital to decrease outbreaks and reduce fatality risks.

The aim of this study was to identify clones of *Acinetobacter baumannii* resistant to carbapenems associated with nosocomial outbreaks and to identify virulence factors that could be involved in dissemination and in fatality risks in three hospitals in Mexico.

## Materials and Methods

### Study Design and Sample Collection

This study was carried out with a single nosocomial infection isolates of *A. baumannii* that were recovered from patients from each of three tertiary referral hospitals in Mexico: Hospital Civil de Guadalajara, Fray Antonio Alcalde (HCG) with 1,000 beds in Guadalajara Jalisco, Mexico; Hospital Regional General Ignacio Zaragoza (HRGIZ), Instituto de Seguridad y Servicios Sociales de los Trabajadores del Estado in Mexico City, Mexico with 360 beds; and the 59 beds Pediatric ward of the Hospital General de México Eduardo Liceaga (HGM-P) in Mexico City, Mexico. The study does not involve humans, it is an *in vitro* study and every bacterial isolates were from a bank of organisms identified in their respective hospital clinical laboratories by VITEK 2 System (bioMerieux, Marcy l’Etoile, France) and Sensititre ARIS^®^ 2X System (TREK Diagnostic Systems Inc., Westlake, OH, United States), confirmed by API20NE (bioMeriux^®^ SA) and by detecting the intrinsic carbapenemase *bla*_OXA–__51__–like_ gene by PCR (Table 1).

**TABLE 1:** Primers used for PCR amplification of β-lactamase and IS*Aba1* genes.

**Gene**	**Primer sequence**	**Amplicon length (bp)**	**T_annealing_ (°C)**	**References**
**Class A β-lactamase**				
*bla*_TEM_	F: ATGAGTATTCAACATTTTCG R: TTACCAATGCTTAATCAGTGAG	861	55	Celenza et al., 2006
*bla*_CTX–M–type_	F: CGCTTTGCGATGTGCAG R: ACCGCGATATCGTTGGT	550	52	Bonnet et al., 2000
*bla*_S__HV_	F: ATGCGTTATATTCGCCTGTGTATT R: TTAGCGTTGCCAGTGCTCGATC	861	60	This study
*bla*_KPC_	F: TCACTGTATCGCCGTCTAGTTCTG R: TTACTGCCCGTTGACGCCCAATC	875	58	This study
**Class B β-lactamase**				
*bla*_VIM_	F: GAGTGGTGAGTATCCGACAGTCAACGAAAT R: AGAGTCCTTCTAGAGAATGCGTGGGAATCT	389	58	This study
*bla*_NDM_	F: GTCTGGCAGCACACTTCCTATCTC R: GTAGTGCTCAGTGTCGGCATCACC	516	58	This study
*bla*_IMP_	F: GCATTGCTACCGCAGCAGAGTCTTTG R: GCTCTAATGTAAGTTTCAAGAGTGATGC	647	58	Alcántar-Curiel et al., 2019
**Class D β-lactamase**				
*bla*_OXA–40–like_	F: TCTAGTTTCTCTCAGTGCATGTTCATC R: CATTACGAATAGAACCAGACATTCC	749	58	This study
*bla*_OXA–51–like_	F: ATGAACATTMAARCRCTCTTACTTA R: CTATAAAATACCTAATTMTTCTAA	825	50	Alcántar-Curiel et al., 2014
IS*Aba1*	F: CACGAATGCAGAAGTTG R: CGACGAATACTATGACAC	548	55	Rosales-Reyes et al., 2017
IS*Aba1*F/OXA-51-likeR	F: CACGAATGCAGAAGTTG R: CTATAAAATACCTAATTMTTCTAA	1380	50	Turton et al., 2006; Rosales-Reyes et al., 2017

### Antimicrobial Susceptibility Testing

The minimum inhibitory concentrations (MICs) were performed according to Clinical and Laboratory Standards Institute guidelines (CLSI, 2018). Dilution method in Müeller Hinton (MH) agar was used to determine MIC to piperacillin, cefepime, imipenem, meropenem, amikacin, and tetracycline. Broth microdilution method was used to determine MICs of colistin (Stendhal Pharma). *Escherichia coli* ATCC 25922 was used as quality control strain.

### Extended Spectrum β-Lactamases and Carbapenemases Screening

Extended Spectrum β-Lactamases (ESBL) production was confirmed with ceftazidime and cefotaxime disks with or without clavulanic acid. The ATCC *Klebsiella pneumoniae* 700603 strain was used to improve detection of ESBL and ATCC *Escherichia coli* 25922 was used as quality control strain. The modified Hodge test (MHT) was used as a screening test to carbapenemase detection in all carbapenem-resistant isolates at the three hospitals (Lee et al., 2001). Metallo-β-Lactamases (MBLs) was determined using carbapenem disks in the presence or absence of EDTA in all carbapenemase producer isolates (Lee et al., 2001). MBL-producing *Pseudomonas aeruginosa* 4677 (Garza-Ramos et al., 2008) and carbapenem-susceptible *A. baumannii* clinical isolate were used as quality control strains.

### PCR Amplification and Gene Sequencing

The presence of genes that encode β-lactamases class A, B and D from Ambler group was screened by PCR (Table 1). Gene amplification of *bla*_OXA–__51__–like_, *bla*_TEM_, and *bla*_CTX–M–__type_ was performed as previously reported (Bonnet et al., 2000; Celenza et al., 2006; Alcántar-Curiel et al., 2014). The primers used to detect *bla*_SHV_ and for the multiplex PCR to detect *bla*_NDM_, *bla*_VIM__,_ *bla*_KPC,_ and *bla*_OXA–__40__–like_ were designed in this work with the primer 3 software (Untergasser et al., 2012). The primer used to detect *bla*_IMP_ in the multiplex PCR were designed previously (Alcántar-Curiel et al., 2019) (Table 1). The presence of the IS*Aba1* promoter sequence and its association with carbapenemase genes was investigated by using IS*Aba-1* (Rosales-Reyes et al., 2017) and IS*Aba1* + *bla*OXA-51-like primers (Turton et al., 2006; Rosales-Reyes et al., 2017). PCR was performed using Master Mix Kit Green GoTaq^®^ (Promega, Madison, WI, United States). The carbapenemases and β-lactamases genes were amplified using the standard conditions: 10 mM of each primer, 1X GoTaq^®^ Green and 3 μL of template DNA extracted by boiling. The amplification conditions for PCR were: initial pre-denaturation at 95°C for 5 min, followed by 35 cycles of denaturation at 95°C for 1 min, annealing at: 50°C for 1 min for *bla*_OXA–__51__–like_, 52°C for 1 min for *bla*_CTX–M–type_, 60°C for 1 min for *bla*_SHV_, 55°C for 1 min for *bla*_TEM_, elongation at 72°C for 1 min, and a final extension step at 72°C for 15 min. The multiplex PCR was performed with 2 mM of each primer and the following reaction parameters: initial pre-denaturation at 95°C for 5 min, 30 cycles of denaturation at 95°C for 30 s, annealing at 58°C for 30 s and elongation at 72°C for 1 min, and a final extension at 72°C for 10 min. Amplified PCR products (except for *bla*_OXA–__51__–like_) were subjected to nucleotide sequencing at the Instituto de Biotecnología, Universidad Nacional Autónoma de México.

### Multilocus Sequence Typing

Multilocus sequence typing (MLST) was performed according to the Oxford scheme for carbapenem-resistant *A. baumannii* isolates that belonged to the same clone from the three hospitals and that caused nosocomial outbreak. The housekeeping genes; *gltA*, *gyrB*, *gdhB*, *recA*, *cpn60*, *gpi*, and *rpoD* were amplified and sequenced as previously described (Bartual et al., 2005). The conditions for PCR reactions were: pre-denaturation at 95°C for 5 min, followed by 35 cycles of denaturation at 95°C for 1 min, annealing at 55°C for 1 min and elongation at 72°C for 1 min, and a final extension step at 72°C for 15 min. Amplified PCR products were subjected to nucleotide sequencing at the Instituto de Biotecnología, Universidad Nacional Autónoma de México. Allelic profiles and sequence types (STs) were identified using BIGSdb software from the PubMLST.org website (Jolley et al., 2018).

### Pulsed-Field Gel Electrophoresis Analysis

The clonal relatedness of *A. baumannii* isolates from the three hospitals was determined by Pulsed-Field Gel Electrophoresis (PFGE) as described previously (Alcántar-Curiel et al., 2014). Genomic DNA was digested with *Apa*I (New England Biolabs, Beverly, MA, United States) overnight at 25°C, followed by PFGE using Gene Path System (Bio-Rad^®^). Classification of all clinical isolates into clones was determined by Tenover criteria (Tenover et al., 1995). The percentage of the similarity profile was calculated using the Dice coefficient (Dice, 1945), with a correlation >85%, and each clinical isolate was considered as member of the same clone.

### Biofilm Production

Clinical isolate of *A. baumannii* were used to test for biofilm formation over polystyrene as previously described with few modifications (Rosales-Reyes et al., 2017). Briefly, one colony was grown on 5 mL of Luria Bertani (LB) broth overnight at 37°C with shacking at 180 RPM. Bacterial cultures were adjusted to 1 × 10^7^ CFUs by mL in LB media and triplicates of 500 μl were dispensed into polystyrene tubes. Following 24 h of static incubation at 37°C, the medium was removed carefully, and the tubes were washed with deionized water. Adherent bacteria were stained with 1% (w/v) crystal violet during 5 min at room temperature. The bacterial-bound crystal violet was dissolved with 1 mL of 100% methanol and quantified by measuring OD_540_ nm. ATCC *A. baumannii* 19606 was used as a positive control. The amount of biofilm formed by the positive control was defined as Biofilm Produced Index (BPI) of 1.0.

### Serum Resistance Assays

Resistance of *A. baumannii* isolates to normal human serum was measured with a conventional assay as reported previously (Rosales-Reyes et al., 2017). Briefly, *A. baumannii* isolates were grown in MH broth. The initial inoculum was adjusted to 1 × 10^7^ bacteria by mL in PBS supplemented with 40% of pooled normal human serum (NHS) (Cedarlane Laboratories Limited, Burlington, ON, Canada) or with heat-inactivated human serum (HIS). Samples were incubated for 3 h at 37°C in static conditions. After the incubation, the number of colony formed units (CFUs) of *A. baumannii* was determined by making serial dilutions 1:10 and plated in MH agar plates. The bacterial activity of the NHS was calculated with the following formula: (CFUs-NHS/CFUs-HIS)^∗^100. As control of NHS activity *Salmonella typhi* 9:12:Vid was used (Gonzalez et al., 1995), results were expressed as percentage of bacterial survival.

### Cell Adherence/Invasion Assays

The adherence/invasion assay was performed as described (Saldaña et al., 2009). Briefly, 8 × 10^5^ A549 cells (human lung tumor cells) were deposited onto 24-well polystyrene plates. Thirty min before infection, the culture media was changed by DMEM without antibiotics. The cells were incubated with *A. baumannii* at a MOI of 10 (with the aim of avoiding cell damage) during 3 h at 37°C. The cells were washed three times with PBS (pH 7.4). Cells were immediately lysed with PBS-Triton X-100 1% to release adhered and intracellular *A. baumannii* from A549 cells. To quantify the adhered and intracellular bacteria (CFUs), serial dilution 1:10 were made and plated onto LB agar plates was performed. These set of experiments were performed in duplicate on 2 different days (*n* = 4). *A. baumannii* ATCC-17961 result was used as a positive control for both adherence and invasion to A549 cells. The number of CFUs obtained from *A. baumannii* 17961 was defined as an adhesion/invasion index of 1.0.

### Cytotoxicity Assays

Monolayers of 3 × 10^5^ A549 cells were infected with *A. baumannii* at a MOI of 100 by 1 h. At 24 h post-infection, supernatants of uninfected or infected cells were assessed to quantify the activity of the cytosolic enzyme: lactate dehydrogenase (LDH; Promega, Madison, WI, United States). The percentage of LDH activity was determined using the following formula: percentage of release = (experimental LDH release – spontaneous LDH release)/(maximal LDH release – spontaneous LDH release) × 100% (Rosales-Reyes et al., 2018).

### Pro-inflammatory Cytokine Release Assays

Monolayers of 3 × 10^5^ A549 cells were infected with *A. baumannii* at a MOI of 100. After infection, the cells were washed and incubated by additional 24 h in DMEM supplemented with gentamicin 100 μg/mL. At 24 h post-infection, the supernatants were used to quantify the presence of TNFα, IL-6, IL-1β by QuantiGlo^®^ ELISA (R&D Systems). The relative light units (RLUs) were determined in a luminometer (Flouroskan Ascent FL by Thermo Fisher^®^), during 1 min, lag time, 0.5 s/well read in an auto gain mode. The data was analyzed with the Ascent Software version 2.4.

### Quantification of Intracellular Superoxide and Nitric Oxide Production

Intracellular superoxide production was quantified as described previously (Rosales-Reyes et al., 2012). Briefly, 3 × 10^5^ A549 cells were infected with *A. baumannii* at a MOI of 100 during 3 h in presence of nitro blue tetrazolium (NBT) 250 μg/mL. The NBT water-insoluble deposits in A549 cells were dissolved with 240 μl of 2 M KOH followed by the addition of 280 μL of DMSO. The superoxide production was quantified at an OD_570_ nm. The nitric oxide production was quantified in the supernatants of uninfected or infected A549 cells with the Griess Reagent System following the manufacturer’s instructions (Promega, Madison, WI, United States).

### Statistical Analysis

The data are shown as mean ± the standard deviation (SD). All statistical analyses were conducted with GraphPad Prism 7.0 software. The unpaired *t-*test was used to compare the SD of two groups. Ordinary one-way ANOVA was used to compare the SD of three groups. Asterisks indicate statistical significance and the *p*-values are denoted as ^∗^*p* < 0.05; ^∗∗^*p* < 0.01 and ^∗∗∗^*p* < 0.001; NS, non-significant.

## Results

### Bacterial Isolates

A total of 202 single nosocomial infection isolates of *A. baumannii* were collected consecutively from January to December 2016 at HCG. The most common site of isolation was respiratory tract 101/202 (50.0%), followed by skin and soft tissue secretions 45/202 (22.2%) (Table 2). These isolates were more frequently derived from the surgical ward 43/202 (21.2%) followed by the medical ward 34/202 (16.8%). Likewise, 42 isolates were collected consecutively from January 2015 to January 2016 at HRGIZ, the most common isolation site was from blood cultures 19/42 (45.2%) followed by skin and soft tissue secretions 12/42 (28.5%). The isolates were more frequent from the intensive care unit 18/42 (42.8%) followed by the medical ward 16/42 (38.0%). Additionally, 8 isolates of *A. baumannii* obtained from January to December 2016 at HGM-P were included; the most common isolation sites were blood cultures 3/8 (37.5%), respiratory tract samples 2/8 (25%) and skin and soft tissue secretions 2/8 (25%).

**TABLE 2:** Characteristics of *A. baumannii* isolated from three Mexican hospitals.

	**Hospital**		
	
**Isolation site**	**HCG No. isolates (%)**	**HRGIZ No. isolates (%)**	**HGM-P No. isolates (%)**
Respiratory tract	101 (50.0)	4 (9.5)	2 (25.0)
Skin and soft tissue secretions	45 (22.2)	12 (28.5)	2 (25.0)
Blood	23 (11.3)	19 (45.2)	3 (37.5)
Central venous access devices	14 (6.9)	5 (11.9)	0
Cerebrospinal fluid	8 (3.9)	2 (4.7)	1 (12.5)
Intra-abdominal	6 (2.9)	0	0
Urine	5 (2.4)	0	0
Total	202	42	8

**Hospital ward**	**No. isolates (%)**	**No. isolates (%)**	**No. isolates (%)**

Surgical ward	43 (21.2)	0	0
Medical ward	34 (16.8)	16 (38.0)	0
Intensive care unit	31 (15.3)	18 (42.8)	0
Nephrology	19 (9.4)	0	0
Infectious diseases unit	15 (7.4)	0	0
Orthopedic/Traumatology	14 (6.9)	6 (14.2)	0
Cardiology	11 (5.4)	0	0
Geriatrics	7 (3.4)	0	0
Pediatrics unit	0	0	8 (100)
Pediatrics intensive care unit	2 (0.9)	2 (4.7)	0
Other sources	26 (12.8)	0	0
Total	202	42	8

### *A. baumannii* Isolates Displayed a Multidrug-Resistant Profile

All *A. baumannii* isolates were assessed to determine the antimicrobial susceptibility to six different antibiotics classes; aminoglycoside, cephems, carbapenems, penicillins, lipopeptides, and tetracyclines (Table 3). The results showed that 197/202 (97.5%) isolates from HCG were imipenem and meropenem resistant. Likewise, 36/42 (85.7%) isolates from HRGIZ were imipenem resistant and 40/42 (95.2%) meropenem resistant. Finally, isolates from HGM-P 7/8 (87.5%) were imipenem and meropenem resistant. The results also showed that most of *A. baumannii* isolates were colistin susceptible. Colistin-resistance were found in 11/202 (5.4%) isolates from HCG, 2/42 (4.8%) from HRGIZ and 1/8 (12.5%) from HGM-P. The carbapenemase activity was identified in 208/242 (85.9%) carbapenem-resistant isolates at the three hospitals and 190/242 (78.5%) of these were MBL producer.

**TABLE 3:** Minimum Inhibitory Concentration data and antimicrobial susceptibility of *Acinetobacter baumannii* from three tertiary care hospitals in Mexico.

**Drug class**	**Antimicrobial agent**	**MIC range CLSI/2018**			**HCG (*n* = 202)**			**HRGIZ (*n* = 42)**			**HGM-P (*n* = 8)**		
					
		**S**	**I**	**R**	**MIC_50_ (μg/mL)**	**MIC_90_ (μg/mL)**	**R%**	**MIC_50_ (μg/mL)**	**MIC_90_ (μg/mL)**	**R%**	**MIC_50_ (μg/mL)**	**MIC_90_ (μg/mL)**	**R%**
Aminoglycosides	Amikacin	≤16	32	≥64	64	>128	76.2	128	128	78.6	>128	>128	87.5
Lipopeptides	Colistin	≤2	–	≥4	0.5	2	5.4	0.5	2	4.8	1	1	12.5
Cephems	Cefepime	≤8	16	≥32	128	>128	88.2	128	>128	100	64	128	75
Carbapenems	Imipenem	≤2	4	≥8	64	128	97.5	128	128	90.5	>128	>128	75
	Meropenem	≤2	4	≥8	128	>128	97.5	128	128	97.6	>128	>128	75
Penicillins	Piperacillin	≤16	32–64	≥128	>256	>256	97.5	>256	>256	100	>256	>256	75
Tetracyclines	Tetracycline	≤4	8	≥16	32	>128	82.2	>128	>128	100	16	≥128	62.5

*HCG, Hospital Civil de Guadalajara. HRGIZ, Hospital Regional General Ignacio Zaragoza, Instituto de Seguridad y Servicios Sociales de los Trabajadores del Estado. HGM-P, Hospital General de México, Pediatric ward. Dilution method in MH agar was used to determine MIC to piperacillin, cefepime, imipenem, meropenem, amikacin, and tetracycline. Broth microdilution method was used to determine colistin MIC.*

### *A. baumannii* Isolates Carried *bla*_OXA–__24_, *bla*_VIM–__1_, *bla*_CTX__–M__–2_, *bla*_TEM__–__1_, *bla*_OXA–__51_ and IS*Aba1* Gene

All *A. baumannii* isolates were confirmed by PCR amplification of *bla*_OXA–__51__–like_ gene. Using a multiplex PCR, we determined the presence of *A. baumannii* producers of carbapenemases from the three hospitals. The results showed that 178/197 (90.3%) isolates from HCG, 24/40 (60.0%) from HRGIZ and 5/7 (71.4%) from HGM-P were carbapenem-resistant that carried *bla*_OXA–__24_ and 1/194 (0.51%) from HCG carried *bla*_VIM__–__1_ gene. It is noteworthy that the distribution of carbapenemase genes among carbapenem-resistant isolates from the three hospitals varied significantly. Additionally, class A β-lactamase genes were assessed in all *A. baumannii* isolates, specifically, the *bla*_TEM__–__1_ gene was detected in 60/202 (29.7%), 29/42 (69.0%), 4/8 (50%) isolates from HCG, HRGIZ and HGM-P respectively. The *bla*_CTX–M__–__2_ gene was detected in only 2/42 (4.76%) isolates from HRGIZ. The *bla*_NDM_, *bla*_KPC_, *bla*_IMP_ and *bla*_SHV_ genes were not found in any of the isolates assessed. In this study we also evaluated the coexistence of carbapenemase and β-lactamase genes. The most frequent resistance profile was *bla*_OXA–__24_ in 125/202 (61.8%) isolates from HCG, *bla*_OXA–__24_ + *bla*_TEM–__1_ in 24/42 (51.7%) isolates from HRGIZ, and *bla*_OXA–__24_ in 4/8 (50%) and *bla*_OXA–__24_ + *bla*_TEM–__1_ in 4/8 (50%) isolates from HGM-P (Table 4).

**TABLE 4:** β-lactamase resistance gene profiles of 252 *A. baumannii* isolates from three Mexican hospitals.

**β-lactamase gene profile**	**HCG (*n* = 202) No. isolates (%)**	**HRGIZ (*n* = 42) No. isolates (%)**	**HGM-P (*n* = 8) No. isolates (%)**
*bla*_OXA–24_	125 (61.8)	0	4 (50)
*bla*_TEM–1_	8 (3.9)	3 (11.9)	0
*bla*_OXA–24_ + *bla*_VIM–1_	1 (0.4)	0	0
*bla*_OXA–24_ + *bla*_TEM–1_	52 (25.7)	24 (57.1)0	4 (50)
*bla*_TEM–1_ + *bla*_CTX–M–2_	0	2 (4.7)	0
None	16 (7.9)	13 (30.9)	0

*HCG, Hospital Civil de Guadalajara; HRGIZ, Hospital Regional General. Ignacio Zaragoza, Instituto de Seguridad y Servicios Sociales de los Trabajadores; HGM-P, Hospital General de México, Pediatric Ward.*

ISAba1was found in 252 *A. baumannii* isolates, but this sequence was not in a collinear form with *bla*_OXA–__51_ gene or *bla*_OXA–__24_ gene.

### Clonal Dissemination of *A. baumannii* Isolates Involved in Nosocomial Outbreaks at Three Hospitals

**FIGURE 1:**
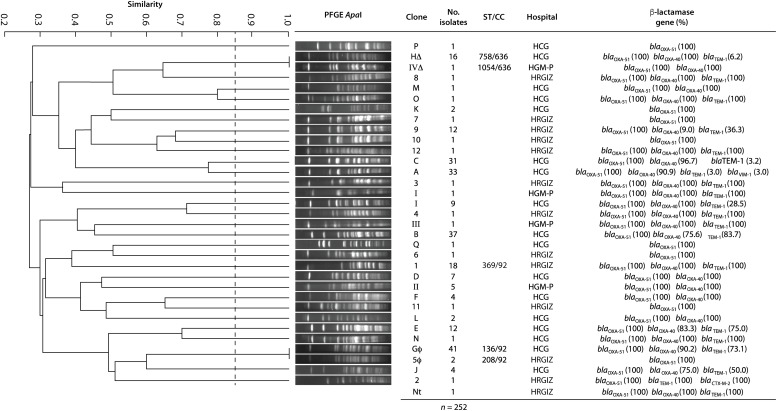
Dendrogram generated from the analysis of 252 *A. baumannii* isolates from three tertiary care Mexican hospitals. A total of 202 isolates from HCG were grouped in 17 different clones, 42 isolates from HRGIZ were grouped in 12 different clones and 8 isolates from HGM-P were grouped in 4 different clones. Δ, φ: Isolates with similar PFGE profile disseminated in the three hospitals. One PFGE profile by clone was used to construct the dendrogram. The dashed line represents the 85% similarity level used in the cluster designation.

**FIGURE 2:**
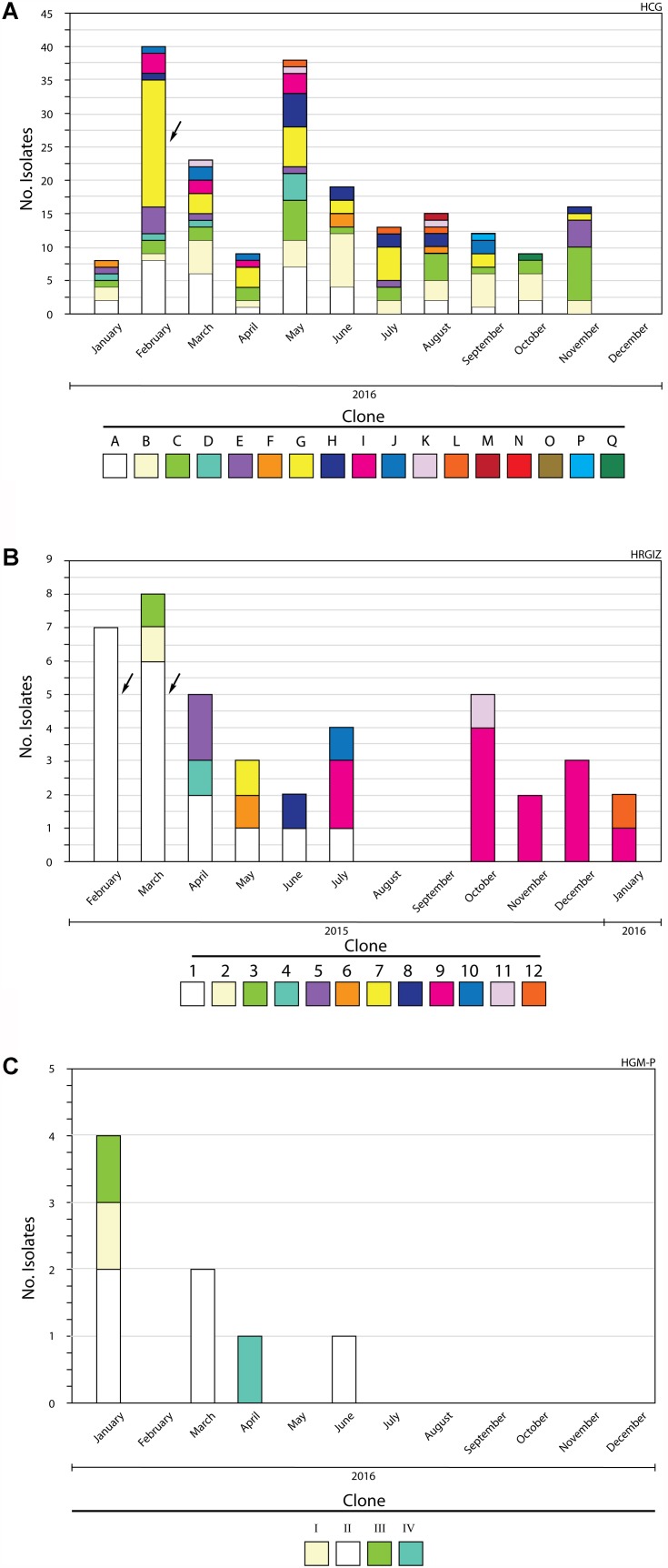
Members of *A. baumannii* clones identified during a 12-month study period. **(A)** Hospital Civil de Guadalajara HCG. **(B)** Hospital Regional General Ignacio Zaragoza, HRGIZ. **(C)** Pediatric ward, Hospital General de México Eduardo Liceaga HGM-P. The arrows show outbreaks of infections caused by *A. baumannii*.

All *A. baumannii* isolates were genotyped by PFGE, every clone received a key in either alphabetical order (HCG), numerical order (HRGIZ) and roman numeral order (HGM-P) respectively (Table 5). Clonal relatedness analysis identified 17 different clones from 202 isolates from HCG. The most frequent clone was the “G” clone with 41/202 (20.2%), followed by the “B” clone with 37/302 (18.3%), “A” clone with 33/2012 (17.2%), and the “C” clone with 31/202 (16.3%) isolates (Figures 1, 2A and Table 5). Clone “G” was involved in a nosocomial outbreak with 19 isolates detected during February 2016 in the intensive care unit and at the medical ward. From HRGIZ 41/42 (97.6%) isolates were grouped in 12 clones but one isolate was non-PFGE typeable. The most abundant clone was the “1” with 18/42 (43.9%) followed by the “9” clone with 12/42 (29.3%) isolates (Figures 1, 2B and Table 5). The clone “1” was involved in a nosocomial outbreak at the UCI during February (7 isolates) March (6 isolates) from 2015. Finally, 8 isolates from HGM-P were grouped in 4 clones with predominance of the clone “II” 5/8 (62.5%) (Figures 1, 2C and Table 5).

**TABLE 5:** Clones identified in 252 *A. baumannii* isolates from three Mexican hospitals and representative isolates selected for the study of virulence factors and immune response.

	**HCG**				**HRGIZ**				**HGM-P**		
					
**Clone**	**MLST Oxford ST/CC**	**No. isolates *n* = 202**	**Selected isolates *n* = 31**	**Clone**	**MLST Oxford ST/CC**	**No. isolates *n* = 42**	**Selected isolates *n* = 31**	**Clone**	**MLST Oxford ST/CC**	**No. isolates *n* = 8**	**Selected isolates *n* = 7**
A		33	4	1	369/92	18	12	I		1	1
B		37	4	2		1	1	II		5	4
C		31	1	3		1	1	III		1	1
D		7	1	4		1	1	IVΔ	1054/636	1	1
E		12	1	5φ	208/92	2	2				
F		4	1	6		1	1				
Gφ	136/92	41	9	7		1	1				
HΔ	758/636	16	1	8		1	1				
I		9	1	9		12	8				
J		4	1	10		1	1				
K		2	1	11		1	1				
L		2	1	12		1	1				
M		1	1	Nt^∗^		1					
N		1	1								
O		1	1								
P		1	1								
Q		1	1								

*Nt: Non-typeable isolate. ^∗^, φ, Δ, isolates belonging to the same clone. ST, sequence type; CC, clonal complex.*

We speculated that some clones could be shared among the three hospitals as observed with PFGE profiles. These results showed that the PFGE profile of clone “G” from HCG was similar to the profile of clone “5” from HRGIZ (Figure 1 and φ in Table 5) and profile of clone “H” from HRGIZ was similar to the profile of clone “IV” from HGM-P (Figure 1 and Δ in Table 5).

### Distribution of the MLST Sequence Types Among *A. baumannii* Isolates

**FIGURE 3:**
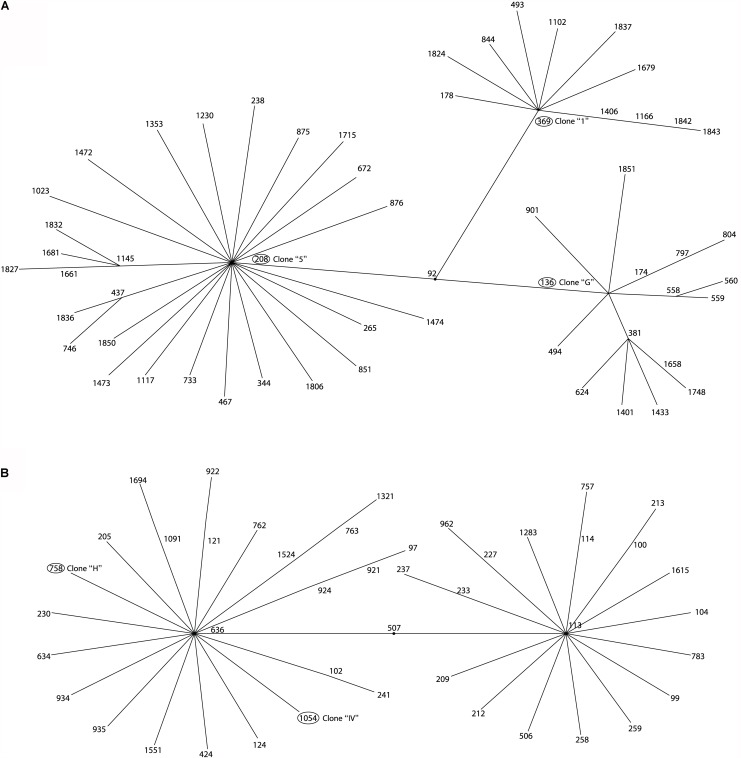
eBURST analysis of *A. baumannii* isolated from three tertiary care hospitals in Mexico. Minimum spanning tree analysis showing the ST-groups of the MLST database where 5 STs of this study are located. **(A)** Related ST groups to ST136, ST369, and ST208 belonging to the clonal complex CC92. **(B)** Related ST groups to ST758 and 1054 belonging to the clonal complex CC636.

The Multi Locus Sequence Typing (MLST) analysis of isolates that belonged to the same clone from the three hospitals showed that the member of the clone “G” from HCG belonged to ST136 and the member of clone “5” from HRGIZ belonged to ST208, these two STs belong to clonal complex 92 (Figures 1, 3A). The member of the clone “H” from HCG belonged to ST758 and member of the clone “IV” from HGM-P belonged to ST1054, these two STs belong to clonal complex 636 (Figures 1, 3B). In addition, the member of the clone “1” involved in the outbreak in the HRGIZ belonged to ST369 of the clonal complex 92. Note that the clone causing outbreak in the HCG was the clone “G” belongs to ST136.

### *A. baumannii* Clinical Isolates Produce Biofilms on an Abiotic Surface

Strains were selected from representative members of each clone from these three hospitals (Table 5). In this selection, we included all clones with single isolates and from clones with several members, included 10–40% of them. With the information of patient mortality associate to *A. baumannii* from HCG and HGM-P we selected a half of all members associated with patient death and a half of improved patients during period hospitalization. Thus, we created a subgroup with 69 isolates; 31 from HCG, 31 from HRGIZ and 7 from HGM-P (Table 5).

**FIGURE 4:**
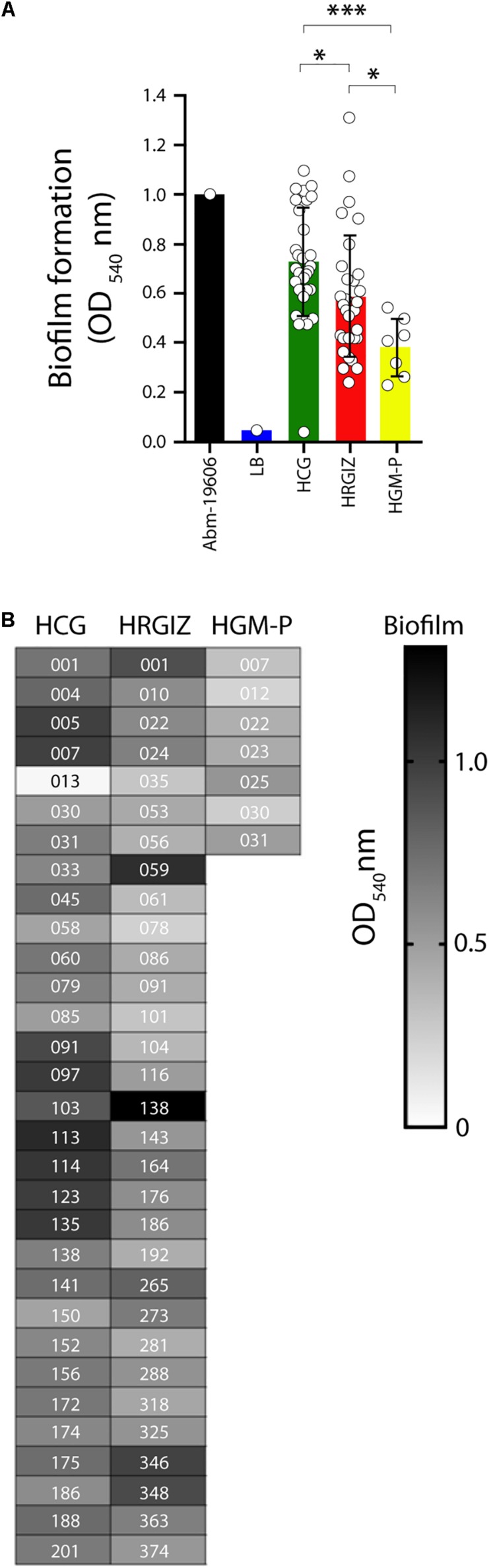
Biofilm production by isolates of *A. baumannii*. Each open circle corresponds to the average of two independent experiments, each one in triplicate (*n* = 6), plotted as the mean ± SD. **(A)** Bars indicate biofilm produced by *A. baumannii* ATCC-19606 by isolates from the HCG, HRGIZ and HGM-P. **(B)** Heat map chart indicates the level of biofilm production by every clinical isolate assessed. Every data indicate the biofilm index produced in relation to *A. baumannii* ATCC-19606. ATCC19606 *A. baumannii* was used as a control strain. ^∗^*p* < 0.05; ^∗∗∗^*p* < 0.001.

All 69 clinical isolates selected were analyzed by their ability to produce biofilms over an abiotic surface. The results showed that isolates from the HCG produce more biofilm than isolates from HRGIZ and HGM-P (Figure 4A). In Figure 4B we show the ability of every isolate to produce biofilm. No differences in biofilm formation were detected when we compared isolates from blood source *vs.* other sources (Supplementary Figure 1A) or when we compared isolates with phenotype of multidrug-resistant (MDR), extensively drug-resistant (XDR), or pandrug-resistant (PDR) (Supplementary Figure 1B).

### *A. baumannii* Survives to Normal Human Serum Activity

**FIGURE 5:**
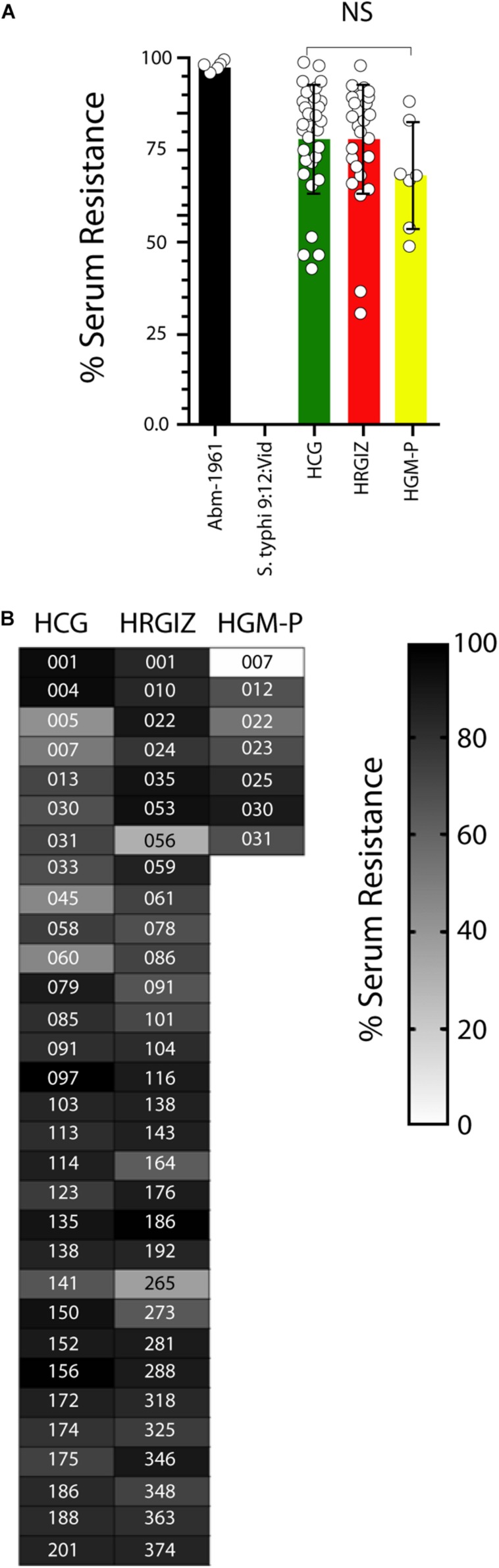
Resistance to normal human serum by isolates of *A. baumannii*. Percentage of bacterial survival is defined as % of serum resistance. Open circles correspond to the average of two independent experiments, each one in triplicate (*n* = 6) plotted as the mean ± SD. **(A)** Bars indicate serum resistance of *A. baumannii* ATCC-17961, *S. typhi*-9:12:Vid and by isolates from the HCG, HRGIZ, and HGM-P. **(B)** Heat map chart indicates the level of serum resistance of each clinical isolate assessed. *A. baumannii*-17961 was used as a strain with resistance to NHS. *S. typhi* was used as a strain with sensitivity to NHS. NS, non-significant.

In this study 28/31 (90.3%) isolates from HCG, 29/31 (93.5%) from HRGIZ and 6/7 (85.7%) from HGM-P survives efficiently to NHS activity (>50–100%) (Figure 5A). In addition, 3/31 (9.7%) from HCG, 2/31 (6.5%) from HRGIZ and 1/7 (14.3%) from HGM-P survive to NHS with less efficiently (>25 – <50%). In Figure 5B we show the ability of every isolate to resist to serum activity. No differences were observed in serum resistance when we compared isolates from blood source vs. other sources (Supplementary Figure 2A) or when we compared isolates with phenotype of MDR, XDR or PDR (Supplementary Figure 2B).

### Adherence/Invasion of *A. baumannii* Clinical Isolates to A549 Cells

**FIGURE 6:**
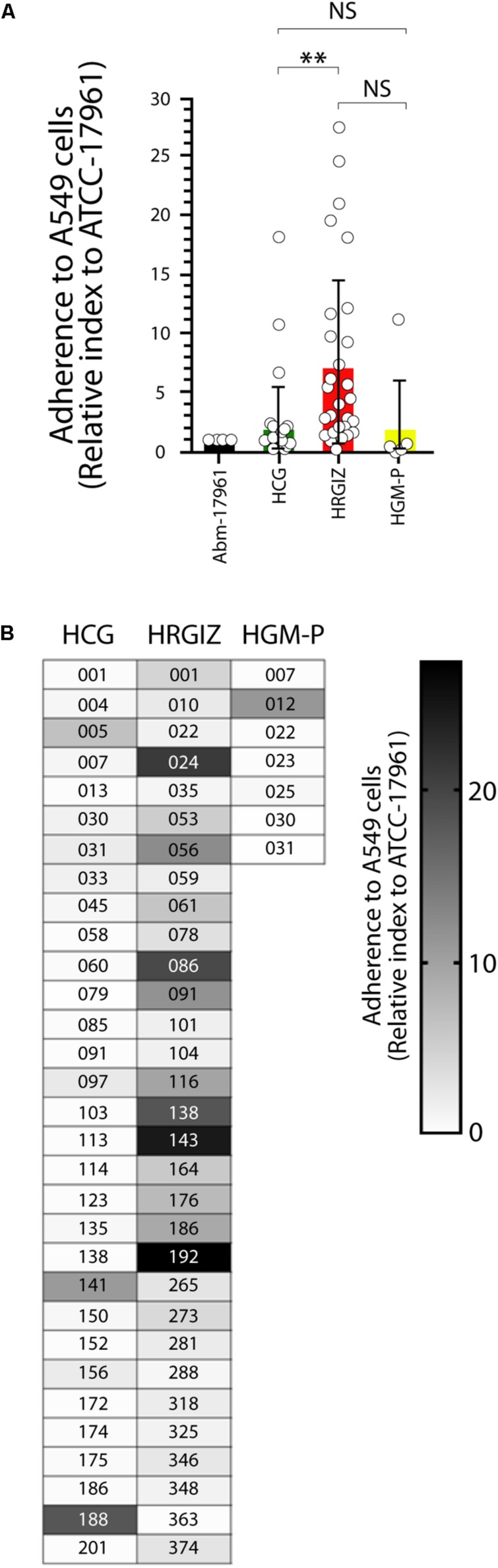
Adherence/invasion of *A. baumannii* to A549 cells. Open circles correspond to each *A. baumannii* clinical isolate grouped by hospital in which was isolated each one in triplicate (*n* = 6). Each bar is plotted as the mean ± SD. **(A)** Bars indicate the adherence/invasion to A549 cells (relative index to *A. baumannii*-17961) of each isolate from the HCG, HRGIZ, and HGM-P. **(B)** Heat map chart indicates the level of adherence/invasion to A549 cells of each clinical isolate assessed. NS, non-significant; ^∗∗^*p* < 0.01.

The results of interactions between the clinical isolates on monolayers of A549 cells showed that the majority of the isolates 30/31 (96.7%) from HRGIZ, only 9/31 (29.0%) from HCG, and 1/7 (14.2%) from HGM-P, were hyper-adherent phenotype (adherence/invasion index of ≥1.0) (Figure 6A). The isolates from the HRGIZ have an increased ability to attach to A549 cells in comparison with isolates from the HCG (Figure 6A). In Figure 6B we show the ability of every isolate to attach A549 cells. No differences were observed in their ability to attach A549 cells when we compared isolates from blood source *vs.* other sources (Supplementary Figure 3A) or when we compared isolates with phenotype of MDR, XDR, or PDR (Supplementary Figure 3B).

### *A. baumannii* Clinical Isolates Induced Cell Death on A549 Cells

**FIGURE 7:**
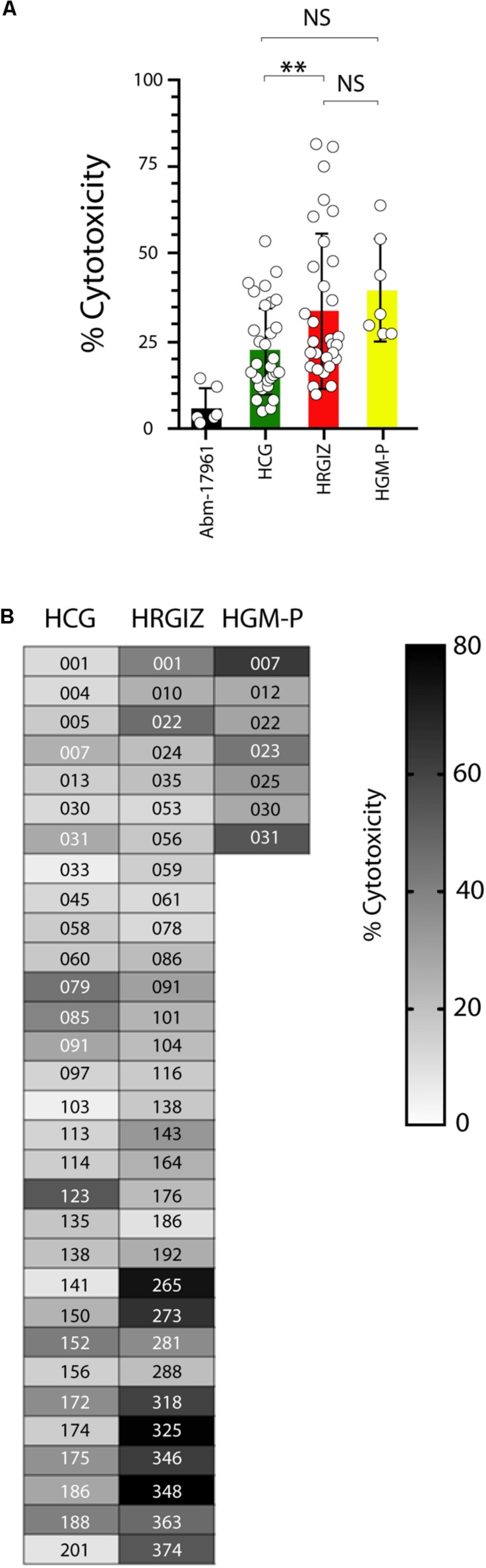
Cytotoxicity Induction by Isolates of *A. baumannii* on A549 cells. Each open circle corresponds to the average of two independent experiments in duplicated (*n* = 4). Each bar is plotted as the mean ± SD. **(A)** Bars indicate the cell death induction (cytotoxicity) of each isolate from the HCG, HRGIZ, and HGM-P on A549 cells. **(B)** Heat map chart indicates the level of cytotoxicity induced by each isolate on A549 cells. NS, non-significant; ^∗∗^*p* < 0.01.

The cell death (cytotoxicity) induction on A549 cells by *A. baumannii* clinical isolates was determined by quantification of the cytosolic Lactate Dehydrogenase (LDH) enzymatic activity on supernatants obtained from infected cells. Results showed that 68/69 (98.5%) isolates induced a variable cytotoxicity (Figure 7A). We also identified that the isolates from HRGIZ were more cytotoxic than isolates from HCG. In Figure 7B we show the ability of every isolate to induce cytotoxicity of A549 cells. No differences in the ability of every isolate from blood source *vs.* other sources (Supplementary Figure 4A) to attach A549 cells were observed. However, isolates with phenotype no multidrug-resistant (NMDR) from HGM-P were more cytotoxic than isolates with XDR, no differences were observed by isolates from HCG and HRGIZ (Supplementary Figure 4B).

### A549 Cells Infected With *A. baumannii* Release of Pro-inflammatory Cytokines

**FIGURE 8:**
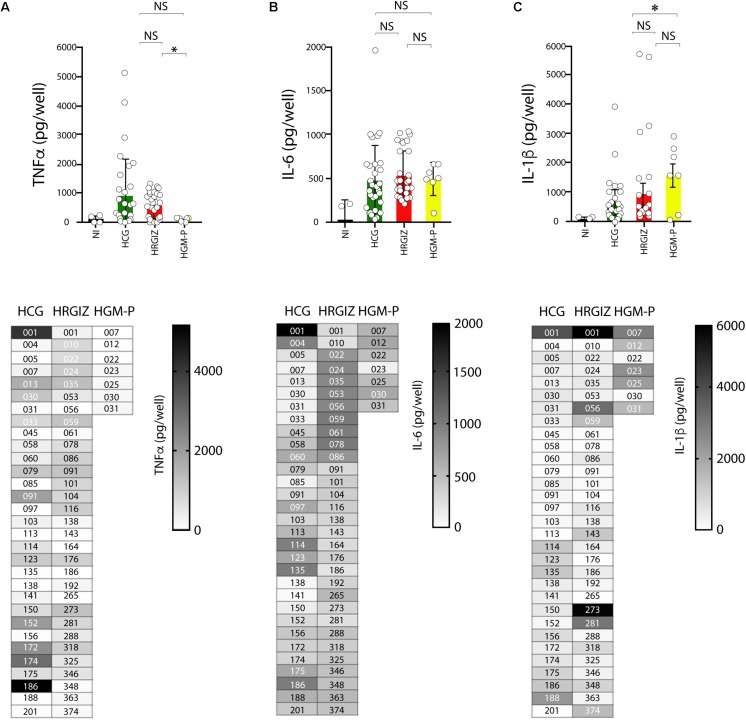
Production of TNFα, IL-6 and IL-1β by A549 infected-cells with isolates of *A. baumannii*. ELISA was used to quantify TNFα **(A)**, IL-6 **(B)**, and IL-1β **(C)** release. Each open circle corresponds to the average of two independent experiments, each one in duplicated (*n* = 4). Each bar is plotted as the mean ± SD. Bars in upper panels indicate the TNFα **(A)**, IL-6 **(B)**, and IL-1β **(C)** released by A549 cells infected with each isolate from the HCG, HRGIZ, and HGM-P. Heat map chart in lower panels indicates the level of cytokine released by A549 infected cells. NI, non-infected. NS, non-significant; ^∗^*p* < 0.05.

*Acinetobacter baumannii* mediate the production of pro-inflammatory cytokines by interaction with A549 cells. TNFα, IL-6 and IL-1β were measured by ELISA on supernatants of infected A549 cells. The results showed that 50/69 (72.4%) isolates mediated the release of 400–5000 pg/mL of TNFα (Figure 8A, upper panels). The results showed that 50/69 (72.4%) isolates mediated the release of 400–5000 pg/mL of TNFα (Figure 8A). A549 cells infected with isolates from HRGIZ released more TNFα than A549 cells infected with isolates from the HGM-P. Also, A549 cells infected with isolates from HGM-P release more IL-1β than A549 cells infected with isolates from the HCG (Figure 8C). No differences in the release of IL-6 by A549 cells infected with isolates from the three hospitals were detected (Figure 8B). In lower panels we show the ability of every isolate to induce the release of TNFα, IL-6 and IL-1β byA549 cells. No differences in the ability of A549 infected cells with every isolate to release TNFα, IL-6 and IL-1β were observed when we compared isolates from blood source *vs.* other sources or when we compared isolates from the three hospitals with phenotype or MDR, XDR, or PDR (Supplementary Figure 5).

### *A. baumannii* Mediates the Oxygen and Nitrogen Reactive Species Production in A549 Cells

**FIGURE 9:**
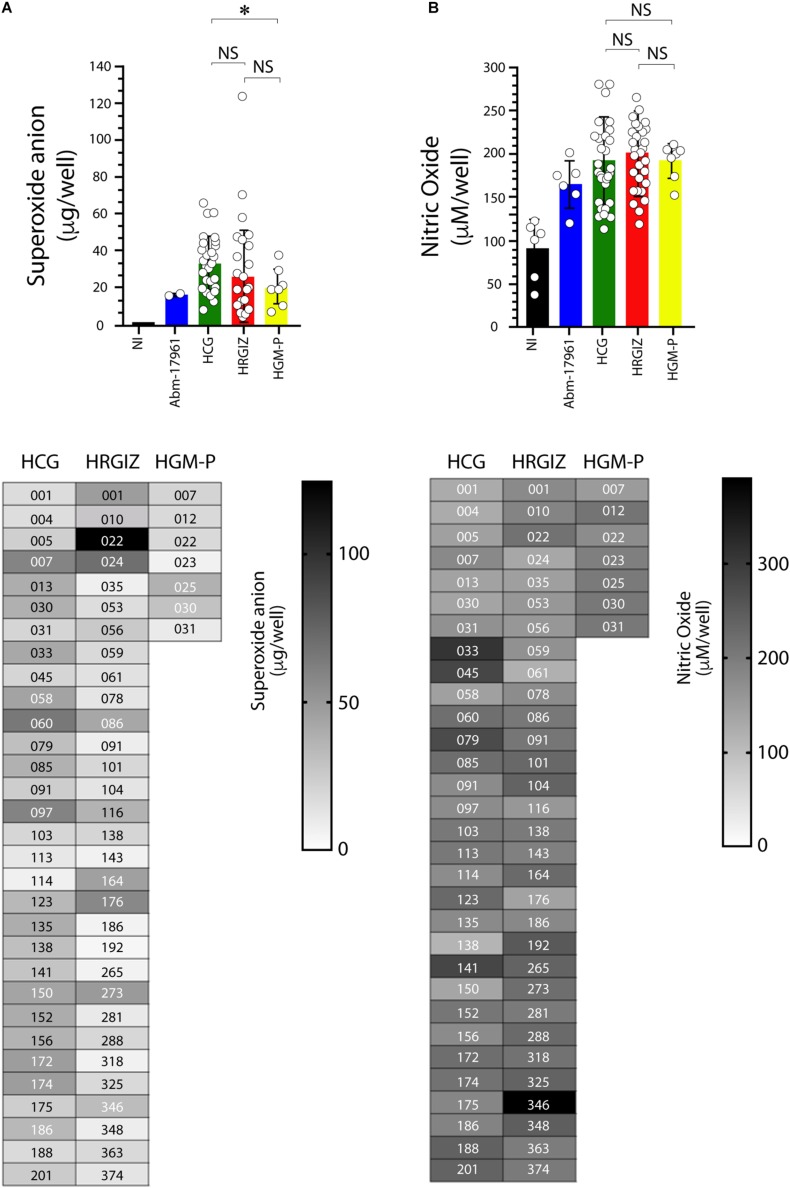
Production of oxygen and nitrogen reactive species by A549 infected-cells with isolates of *A. baumannii*. The oxygen reactive species (superoxide anion) was quantified at 3 h post-infection **(A)** and the nitrogen reactive species (nitric oxide) at 24 h post-infection **(B)**. Each open circle corresponds to the average of two independent experiments, each one in duplicated (*n* = 4). Each bar is plotted as the mean ± SD. Bars in upper panels indicate the superoxide anion **(A)** and nitric oxide **(B)** production by A549 cells infected with each isolate from the HCG, HRGIZ, and HGM-P. Heat map chart in lower panels indicates the level of superoxide anion and nitric oxide production by A549 infected cells. *A. baumannii*-17961 was used as a control strain of these experiments. NI, non-infected. NS, non-significant; ^∗^*p* < 0.05.

The quantification of oxygen reactive species production during the interaction between selected *A. baumannii* clinical isolates with A549 cells showed that 53/69 (76.8%) isolates induced the production of superoxide anion (Figure 9A) In contrast, 16/69 (23.1%) were negative for superoxide anion production. The result shows that A549 infected cells with isolates from HCG mediates a better production of superoxide anion than HGM-P.

Isolates from the HCG induce more production of superoxide anion by infected A549 cells than HGM-P (Figure 9B).

The quantification of nitrogen reactive species production by A549-infected cells showed that 67/69 (97.1%) isolates induce the nitric oxide production (Figure 9B). Isolates from the three hospitals induce similar production of nitric oxide by infected A549 cells. In lower panels we show the ability of every isolate to induce the release of superoxide anion (Figure 9A) or nitric oxide (Figure 9B). No differences were observed in the ability to produce superoxide anion or nitric oxide when we compared isolates from blood source *vs.* other sources or when we compared isolates from the three hospitals with phenotype or MDR, XDR, or PDR (Supplementary Figure 6).

## Discussion

*Acinetobacter baumannii* is a multidrug resistant bacteria that has become a critical threat worldwide due to its ability to survive in the hospital environment and to cause frequent nosocomial outbreaks (Mancilla-Rojano et al., 2019). In this study we characterized 252 nosocomial infections isolates of *A. baumannii* from three tertiary care hospitals in Mexico. The results demonstrated that the majority of these clinical isolates were multidrug-resistant to the commonly prescribed antibiotics for *A. baumannii* infections. The analysis shows a high prevalence of resistance to amikacin (76.2–87.5%), cefepime (75.0–97.5%), piperacillin (75–97.5%), imipenem (75–97.5%), meropenem (75–97.5%), and susceptible only to colistin (4.8–12.5%). The resistance prevalence data for the antibiotics tested in this study are in keeping with the data reported (2011 –2016) by the Organization for Economic Co-operation and Development where Mexico is a member (Xie et al., 2018). Additionally, the comparative analysis of carbapenem-resistance rate in *A. baumannii* isolates from HCG previously reported by our group (Alcántar-Curiel et al., 2014) with the data in this study, showed an increase of imipenem resistance from 71.3% in 2004 – 2011 to 97.5% and for meropenem from 84% in 2004 – 2011 to 97.5%. We observed a slight decrease in carbapenems resistance rate in *A. baumannii* isolates from the Pediatrics ward of the HGM, compared with the data that we reported previously (Rosales-Reyes et al., 2017), imipenem resistance rate decreased from 89.2% in 2014 to 75% in this study and meropenem resistance decreased from 96.4% in 2014 to 75% in this study.

The OXA-24 carbapenemase, a member of the OXA-40-like family, was identified in the great majority of *A. baumannii* with resistance to carbapenems; 177/197 (89.8%) were from HCG, 18/49 (55.0%) from HRGIZ and 4/6 (66.6%) from HGM-P which is reported for the first time in Mexico. The detection of the *bla*_OXA–__24_ gene was reported recently in Brazil (de Azevedo et al., 2019). Although in previous studies, including this one, we have identified OXA-40-like carbapenemases in *A. baumannii* (Alcántar-Curiel et al., 2014; Rosales-Reyes et al., 2017), we can’t rule out that these isolates carry other enzymes type OXA-23, OXA-58, OXA-143. Interesting, only one isolate from HCG was carrier of *bla*_OXA–__24_ and *bla*_VIM–__1_ carbapenemase genes. These results suggest that the high percentage of MBL producing isolates identified (78.5%) may be due to presence of other MBL carbapenemases such as IMI, NMC, GES, SIM, SPM, and GIM that were not investigated.

Different mechanisms are also associated with resistance to carbapenems, therefore we assumed that 12/197 (6.0%) of isolates from HCG with resistance to carbapenems and 11/36 (30.5%) from HRGIZ (Table 4) may express others type of carbapenemases, activity of efflux pumps and/or the loss of porins that we didn’t investigate.

Additionally, *bla*_TEM–__1_ and in a smaller proportion *bla*_CTXM–__1_ which belong to β-lactamases of class A, were also detected. The presence of these β-lactamases has been previously associated with the resistance to the third and fourth generation of cephalosporins and carbapenems and they are also associated with other β-lactamases from other families causing multi-drug resistance phenomena (Zhou et al., 2015).

The clonal relationship among the clinical isolates of *A. baumannii* was determined using the PFGE typing. The results showed that in HCG the most prevalent clone was “G” with 41/202 isolates and was associated with a nosocomial outbreak in February 2016. The comparative analysis between the PFGE profiles of *A. baumannii* isolates from HCG of this study and those previously reported in the same hospital (Alcántar-Curiel et al., 2014), showed that clone “G” profile is similar to the clone previously named “22” that was the most prevalent in the period 2005–2011 and that also caused a nosocomial outbreak. This result indicates that the clone “G” or “22” carrying *bla*_OXA–__24_ gene has disseminated successfully within the different hospital wards for extended periods of time. Because clone “G” was associated with high mortality 19/41 (46.3%), it should be considered as being a high-risk multi-drug-resistant clone. With regard to the isolates from HRGIZ, we detected that the most prevalent clone was “1” with 18/42 isolates and was associated with a nosocomial outbreak from February to March 2015. This clone belonged to ST369 previously reported in *A. baumannii* isolates during period 2005–2011 in northern Mexico (Bocanegra-Ibarias et al., 2015). With the HGM-P isolates we did a comparative analysis between the PFGE profiles in this study with isolates from a previously work reported in the same hospital (Rosales-Reyes et al., 2017). The results showed that the three clones prevalent in 2014 did not remain in this hospital.

The remarkable number of isolates that belonged to the same clone (clones “G,” “B,” “A,” “C,” “H” from HCG, clones “1,” “9” from HRGIZ and clone “IV” from HGM-P) suggests that the dissemination of these clones could be implicated in the resistance to carbapenems of *A. baumannii* strains detected in this study.

One of the most interesting data of the clonal relation of these isolates from three hospitals was the detection of two different profiles of PFGE that seems to be similar among members of clones from hospitals located at great distances from each other; clones “G” and “5” were isolated between hospitals that are located at a distance of 562 km and clones “H” and “IV” were isolated between hospitals that are at a distance of 540 km. These results demonstrated the spread of successful clones of *A. baumannii bla*_OXA–__24_ among different hospitals from different cities in Mexico. The results suggest that the endemicity of multidrug-resistant *A. baumannii* in HCG and HRGIZ is related to: (a) its ability to survive in clinical settings due to antibiotic selection pressure of this environment, (b) its dissemination and coexistence of multiple clones (17 clones in HCG, 14 clones in HRGIZ, in the different hospital areas) and (c) to the spread of carbapenem-resistant *A. baumannii* clones (clone “G” from HCG and clone “1” from HRGIZ) associated with nosocomial outbreaks. This ability to persist and become endemic has been reported previously in *A. baumannii* clinical isolates in Guadalajara, Mexico (Morfin-Otero et al., 2013).

MLST showed that similar clones of *A. baumannii* detected in three Mexican hospitals belonged to different STs. With eBurst, we analyzed the distribution of the STs of one member of these clones with a similar PFGE profile and showed a dispersive phenomenon among them but with a similar genetic background: For example, clone “G” and “5” corresponded to ST136 and ST208 respectively but to the same clonal complex CC92, and clones “H” and “IV” corresponded to ST758 and ST1054 respectively and to clonal complex CC636.

It is important to highlight that these isolates belonging to clones “G”, “H” and “IV” carried the *bla*_OXA–__24_ gene, these results suggest that the dissemination of *bla*_OXA__–__24_ genes in *A. baumannii* isolates with different STs. These findings confirm that OXA-24 is not restricted to one ST, which is not that surprising since OXA-24 is an acquired mechanism of resistance.

In this work we identified ST758 in one isolate from Guadalajara Jalisco, Mexico. This ST has been previously identified in four hospitals in Mexico City, Mexico (Tamayo-Legorreta et al., 2014; Graña-Miraglia et al., 2017; Mancilla-Rojano et al., 2019). ST208 was identified in this work in a hospital in Mexico City, this ST has been also previously reported in isolates from San Luis Potosí, Mexico (Tamayo-Legorreta et al., 2016). ST369 was identified in this work in one isolate from a hospital in Mexico City that has been also detected in Nuevo León, Mexico (Bocanegra-Ibarias et al., 2015). These results demonstrate that the ST758, ST369, and ST208 were distributed in different provinces in Mexico. The STs and the eBurst arithmetic showed that CC92 and CC636 were the clonal complexes disseminated in these three hospitals in Mexico. The ST758 and ST1054 have been described previously and belong to the Ibero American complex CC636, which has been considered a high-risk clone due to association with multidrug resistance phenotype (Graña-Miraglia et al., 2017; Mancilla-Rojano et al., 2019). Likewise, ST208 and ST136 previously reported belong to CC92, the largest and most widely disseminated complex worldwide that has been associated with isolates with ability to acquire resistance determinants and survive in the nosocomial environment (Ruan et al., 2013; Tada et al., 2013). To our knowledge, this is the first description of ST136 and ST1054 in Mexico and demonstrates the emergence of strains belonging to different ST number, suggesting that a new epidemiological shift has occurred.

We previously demonstrated that *A. baumannii* infections could be associated to higher rates of mortality that ranging 28.2–52.8% (Rosales-Reyes et al., 2016, 2017) these data are in line with reports of other countries (Jamulitrat et al., 2009; Lee et al., 2015). The virulence factors associated with persistence of infections in the hospital environment was evaluated by the ability of *A. baumannii* to form biofilms on an abiotic surface. The results showed that 98.5% (68/69 isolates) of the clinical isolates were biofilm producers over polystyrene. Biofilm production was not associated with the clonal spread in hospitals (Hu et al., 2016). Also, no association was detected with any antibiotic-resistance profile, which could point to the persistence of *A. baumannii* in the hospital environment (Supplementary Figure 1). The majority of *A. baumannii* were attached to A549 cells, 58.0% (40/69) of the clinical isolates presented an adherent phenotype. Remarkably, isolates from the HRGIZ presented a high adherent phenotype (Figure 6B). *A. baumannii* attachment to A549 cells could be associated with the expression of the outer membrane protein 33 (Omp33) (Smani et al., 2013), OmpA (Choi et al., 2008b; Smani et al., 2012), Bap (Brossard and Campagnari, 2012) or with the presence of fibronectin binding proteins (FBPs) (Smani et al., 2012).

*Acinetobacter baumannii* interaction with A549 cells induced variable cytotoxicity, interestingly; the 14.5% (10/69) induced more than 50% of cytotoxicity on A549 cells. In contrast to our results, Lazaro-Diez et al. (2016) reported that all *A. baumannii* strains failed to induce any cytotoxic effect on A549 cells, in this way, it’s possible that these isolates reported are not adherent.

In our study, we confirmed that during the *A. baumannii* interaction with A549 cells, the infected cells can release pro-inflammatory cytokines that may contribute to inflammatory immune response. Interestingly, all clinical isolates assessed were able to mediate the TNFα, IL-6 and IL-1β release in different magnitudes. For example, the majority of isolates induced the release of IL-6, in contrast, clones from HCG induced the release of TNFα more efficiently that the clones from HRGIZ or HGM-P. Our results are according to previous data showing that the early recruitment of neutrophils via induction of pro-inflammatory cytokines into the lung is critical for initiating an efficient host innate immune defense against respiratory *A. baumannii* (Qiu et al., 2009) and that the pro-inflammatory cytokines are produced in high levels on a murine model or by murine macrophages activated with LPS from *A. baumannii* (Korneev et al., 2015; Rosales-Reyes et al., 2017). Others studies have demonstrated that during the *A. baumannii* interaction with A549 cells, infected cells produce variable amounts of TNFα or IL-6 (Smani et al., 2011). Interestingly, the release of TNFα and IL-6 by infected cells requires the expression of TLR4 (Kim et al., 2013). Nevertheless, *in vivo*, the optimal production of TNFα is dependent of the TLR9 expression (Noto et al., 2015).

In addition, the inflammatory environment induced during *A. baumannii* increases when infected cells produced oxygen and nitrogen reactive species (Smani et al., 2011). Our results indicate that the majority of the clinical isolates assessed were able to mediate the production of oxygen and nitrogen reactive species by A549 infected cells, these results suggest an important contribution in the inflammatory response during *A. baumannii* infections.

Finally, *A. baumannii* is frequently associated to bacteremia, the most significant infection caused by this bacterium (Cisneros and Rodriguez-Bano, 2002). This may be due in part to the *A. baumannii* ability to survive through their ability to resist to the complement activity (Antunes et al., 2014; García-Patiño et al., 2017). In a previous study we reported that a significant proportion of clinical *A. baumannii* isolates assessed were resistant to killing by the NHS activity (Rosales-Reyes et al., 2017). The results presented in this study are consistent because the majority of the clinical isolates assessed from the three hospitals were resistant to NHS, it’s ability may be due to the expression of bacterial factors like OmpA (Kim et al., 2009) or LPS (Garcia et al., 2000).

## Conclusion

Our study demonstrated a high prevalence of *A. baumannii* with multidrug-resistance including carbapenems in three Mexican tertiary care hospitals. In this study we identified the *bla*_OXA–__24_ gene for the first time in Mexico. The results also revealed a genetic diversity among STs that are involved in outbreaks identified in each hospital and during dissemination among hospitals. We report for the first time in Mexico the presence of ST136 and ST1054. The propagation of multidrug-resistant clones of *A. baumannii* among the hospitals could be associated to the dissemination of the antibiotic resistance genes leading to conventional failure of common antibiotic therapy treatments. Dissemination of clones with ability to survive the lytic effects of normal human serum, may allow this bacterium to multiply in blood and promote inflammatory immune responses in seriously ill patients. Taken together, our findings demonstrated the presence of several phenotypes associated to *A. baumannii* virulence factors that might contribute to the establishment of nosocomial infections and become important risk factors for critically ill hospitalized patients.

## Data Availability

The raw data supporting the conclusions of this manuscript will be made available by the authors, without undue reservation, to any qualified researcher.

## Ethics Statement

This study was submitted and approved by the Facultad de Medicina, Universidad Nacional Autónoma de México Ethics and Research Committee under the register numbers 107/2013 and 084/2016 and by the Hospital Civil de Guadalajara Ethics Committee under the register number 046/16.

## Author Contributions

MA-C conceived, designed, and supervised the study, analyzed the data, and wrote and edited the manuscript. RR-R conceived and designed the study, supervised and performed the experiments, analyzed the data, and wrote and edited the manuscript. MJ-Q, CG-V, JF-V, and JT-T performed the experiments, analyzed the data, and revised the manuscript. PG-V, AL-H, DV-V, ML-Á, and ME-S performed the experiments. SG-C, RM-O, ER-N, and JS-P analyzed the data and revised the manuscript.

## Conflict of Interest Statement

The authors declare that the research was conducted in the absence of any commercial or financial relationships that could be construed as a potential conflict of interest.

## Supplementary Material

FIGURE S1 Biofilm production by isolates of *A. baumannii*. Each open circle corresponds to the average of two independent experiments, each one in triplicate (*n* = 6), plotted as the mean ± SD. **(A)** Biofilm production by isolates from each hospital comparing blood source vs other sources and **(B)** by its phenotype of MDR, XDR, PDR or NMDR. NS, non-significant.

FIGURE S2 Resistance to normal human serum by isolates of *A. baumannii*. Percentage of bacterial survival is defined as % of serum resistance. Open circles correspond to the average of two independent experiments, each one in triplicate (*n* = 6), plotted as the mean ± SD. **(A)** Bars indicate the resistance to normal human serum by isolates from each hospital compared by blood source vs other sources and **(B)** by its phenotype of MDR, XDR, PDR or NMDR. NS, non-significant.

FIGURE S3 Adherence/invasion by isolates of *A. baumannii* to A549 cells. Open circles correspond to the average of two independent experiments by triplicate (*n* = 6), plotted as the mean ± SD. **(A)** Bars indicate the adherence/invasion by isolates from each hospital comparing blood source vs other sources and **(B)** by its phenotype of MDR, XDR, PDR or NMDR. NS, non-significant.

FIGURE S4 Cytotoxicity induction by isolates of *A. baumannii* o*n* A549 cells. Each open circle corresponds to the average of two independent experiments by triplicate (*n* = 6), plotted as the mean ± SD. **(A)** Bars indicate the cell death induction (cytotoxicity) of each isolate from each hospital comparing blood source vs other sources and **(B)** by its phenotype of MDR, XDR, PDR or NMDR. NS, non-significant; significant *p* is indicated.

FIGURE S5 Production of TNFα, IL-6 and IL-1β by A549 infected-cells with isolates of *A. baumannii*. Each open circle corresponds to the average of two independent experiments in duplicated (*n* = 4), plotted as the mean ± SD. **(A)** Bars indicate the TNFα, **(C)** IL-6 and **(E)** IL-1β released by A549 infected-cells with each isolate from each hospital comparing blood source vs other sources and **(B)** the TNFα, **(D)** IL-6 and **(F)** IL-1β released by A549 comparing phenotypes of MDR, XDR, PDR or NMDR. NS, non-significant.

FIGURE S6 Production of oxygen and nitrogen reactive species by A549 infected-cells with isolates of *A. baumannii*. Each open circle corresponds to the average of two independent experiments in duplicated (*n* = 4), plotted as the mean ± SD. **(A)** Superoxide anion or **(C)** Nitric oxide production by A549 infected-cells with each isolate from each hospital were compared by blood source vs other sources and **(B)** Superoxide anion or **(D)** Nitric oxide production by A549 infected-cells by its phenotype of MDR, XDR, PDR or NMDR. NS, non-significant.
