# Supplementary figures and images for: Carbapenem-Resistant Acinetobacter baumannii in Three Tertiary Care Hospitals in Mexico: Virulence Profiles, Innate Immune Response and Clonal Dissemination
Source: Front Microbiol. 2019 Sep 20;10:2116. doi: 10.3389/fmicb.2019.02116 (PMC6764332; doi:10.3389/fmicb.2019.02116)

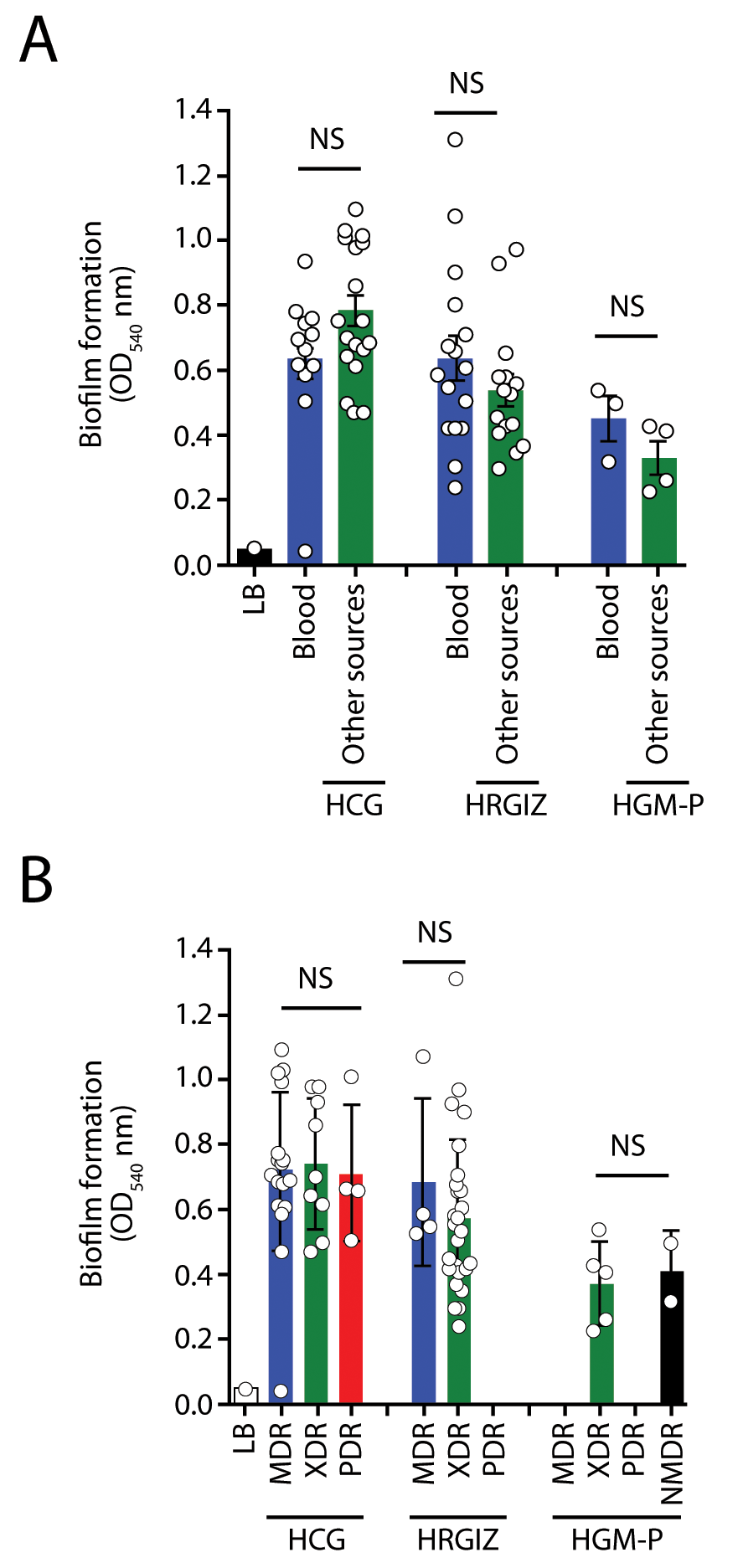

Supplement: FIGURE S1 — Biofilm production by isolates of A. baumannii. Each open circle corresponds to the average of two independent experiments, each one in triplicate (n = 6), plotted as the mean ± SD. (A) Biofilm production by isolates from each hospital comparing blood source vs other sources and (B) by its phenotype of MDR, XDR, PDR or NMDR. NS, non-significant. [file Image_1.TIF]

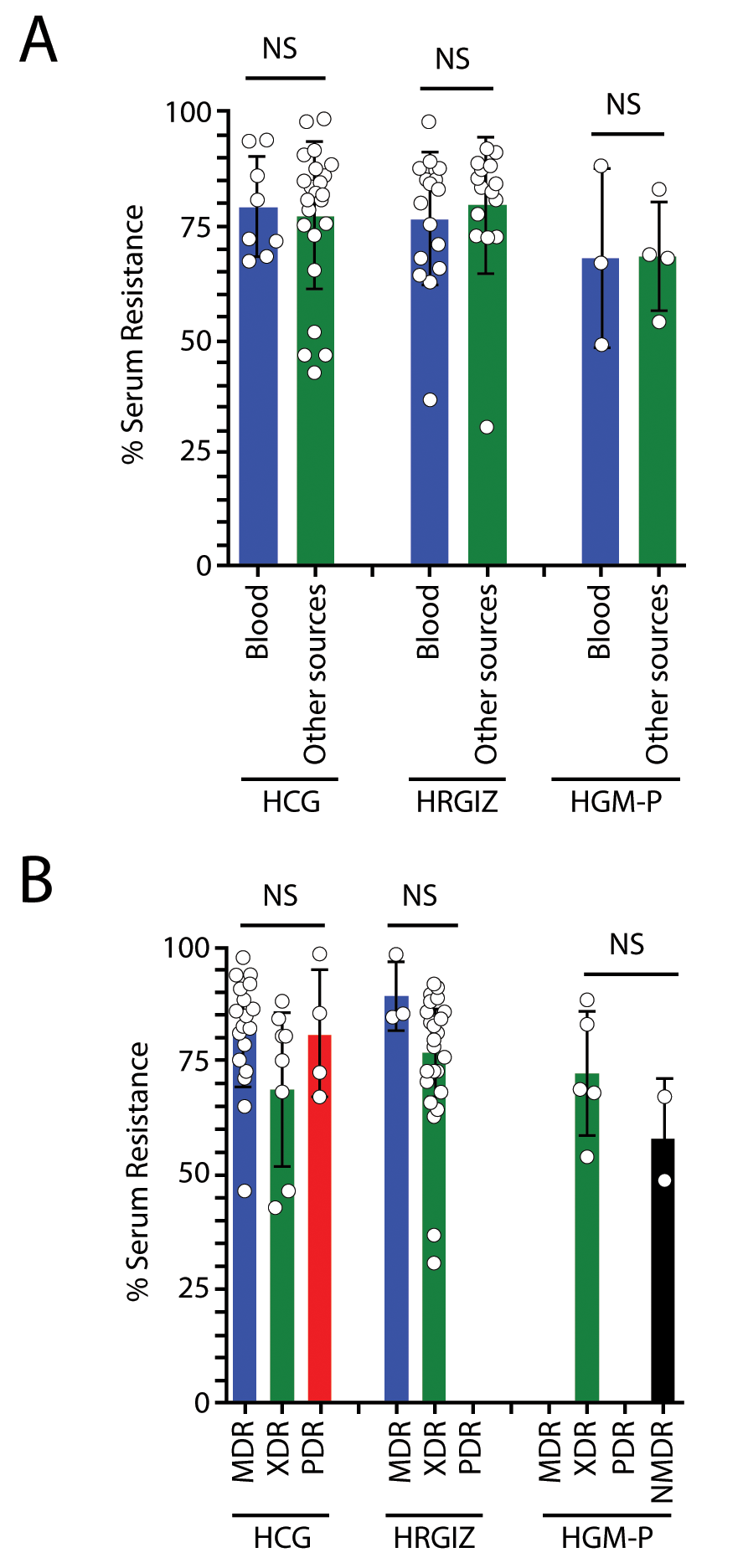

Supplement: FIGURE S2 — Resistance to normal human serum by isolates of A. baumannii. Percentage of bacterial survival is defined as % of serum resistance. Open circles correspond to the average of two independent experiments, each one in triplicate (n = 6), plotted as the mean ± SD. (A) Bars indicate the resistance to normal human serum by isolates from each hospital compared by blood source vs other sources and (B) by its phenotype of MDR, XDR, PDR or NMDR. NS, non-significant. [file Image_2.TIF]

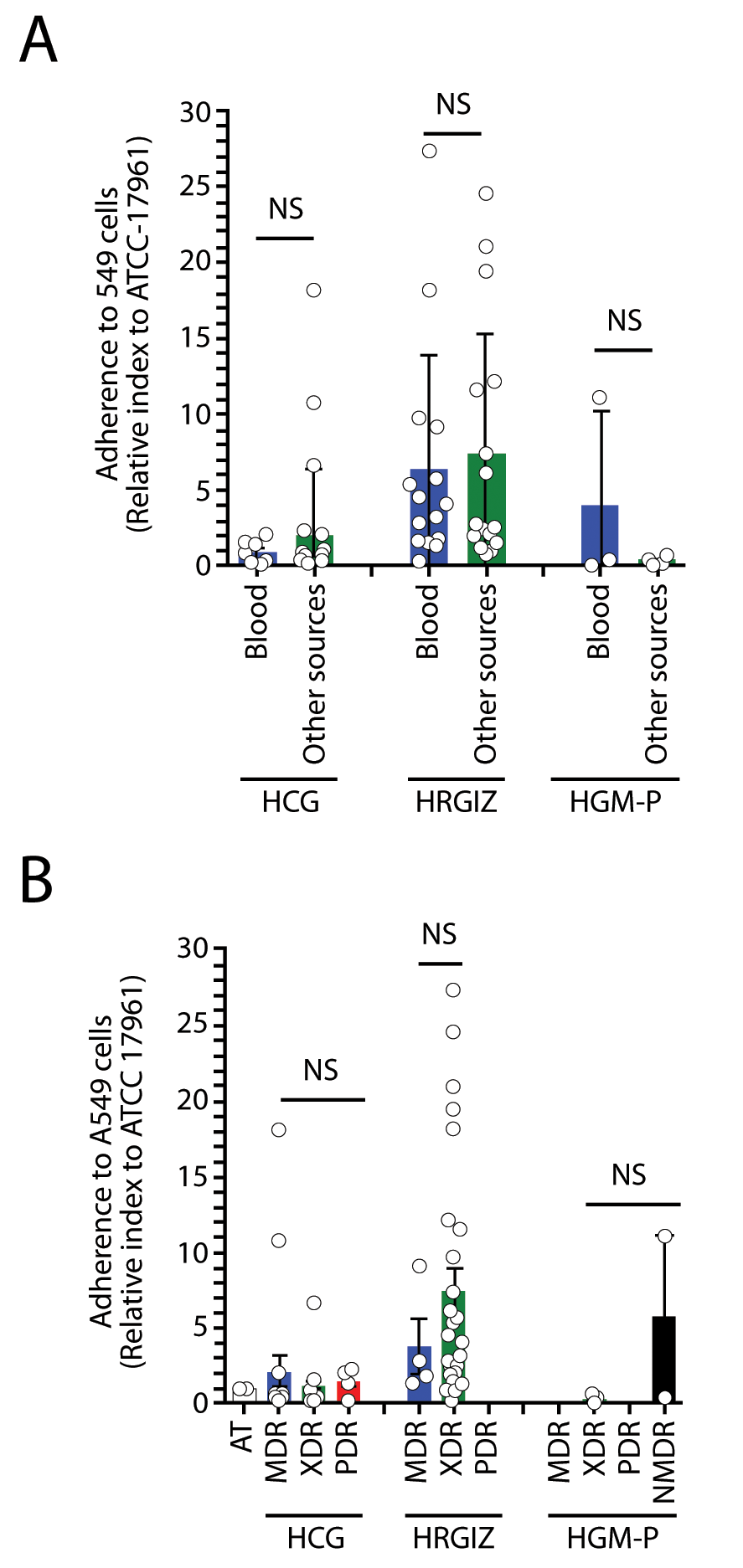

Supplement: FIGURE S3 — Adherence/invasion by isolates of A. baumannii to A549 cells. Open circles correspond to the average of two independent experiments by triplicate (n = 6), plotted as the mean ± SD. (A) Bars indicate the adherence/invasion by isolates from each hospital comparing blood source vs other sources and (B) by its phenotype of MDR, XDR, PDR or NMDR. NS, non-significant. [file Image_3.TIF]

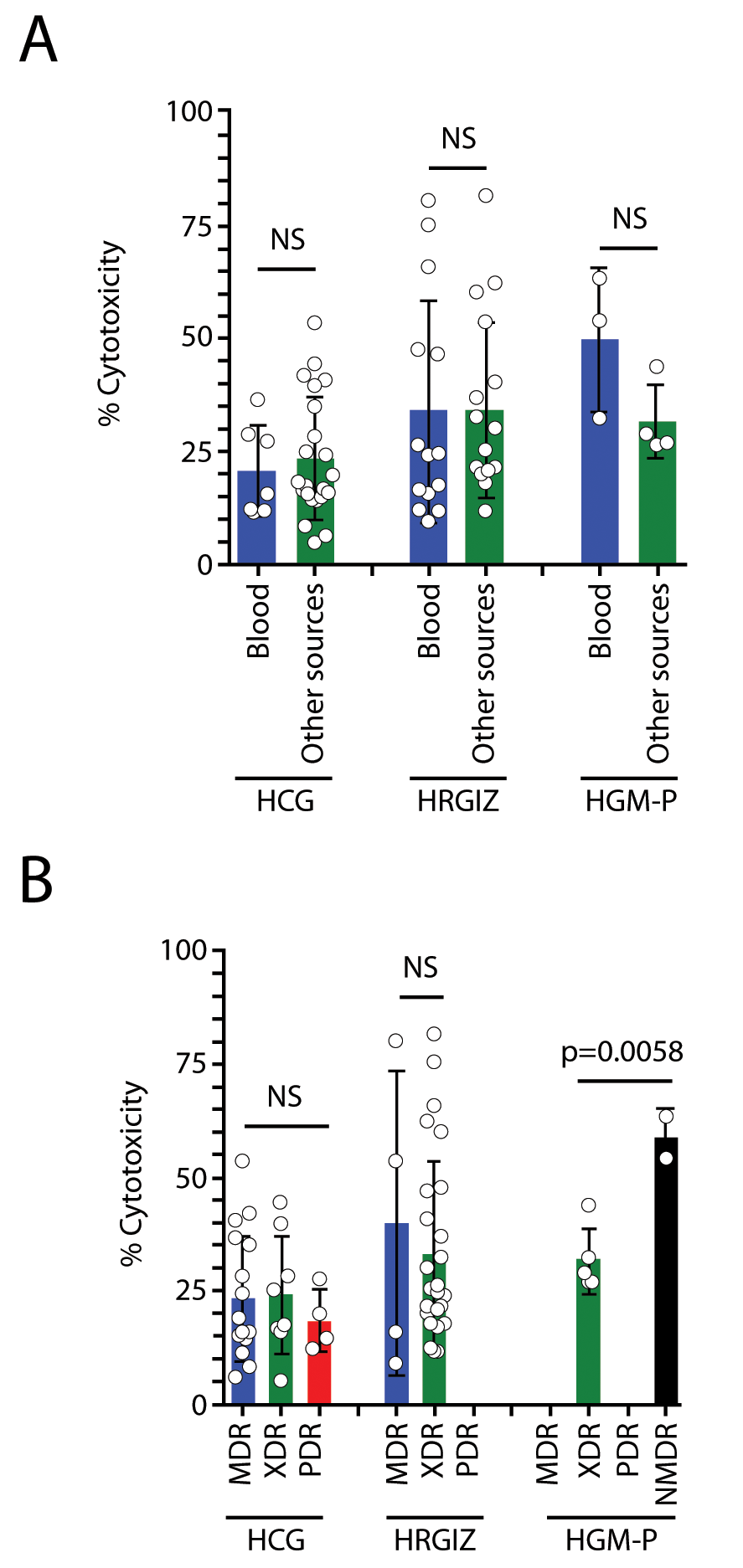

Supplement: FIGURE S4 — Cytotoxicity induction by isolates of A. baumannii on A549 cells. Each open circle corresponds to the average of two independent experiments by triplicate (n = 6), plotted as the mean ± SD. (A) Bars indicate the cell death induction (cytotoxicity) of each isolate from each hospital comparing blood source vs other sources and (B) by its phenotype of MDR, XDR, PDR or NMDR. NS, non-significant; significant p is indicated. [file Image_4.TIF]

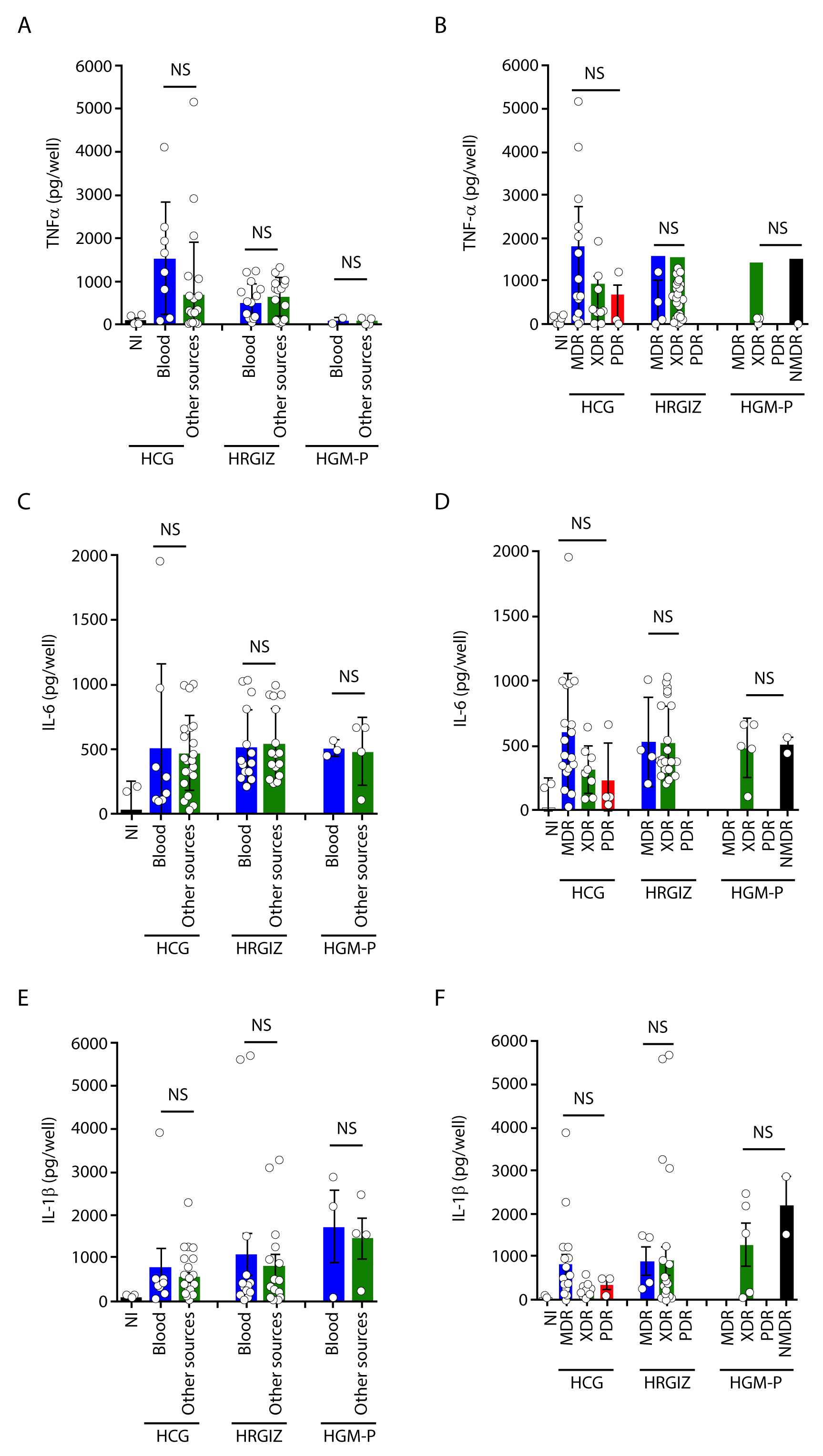

Supplement: FIGURE S5 — Production of TNFα, IL-6 and IL-1β by A549 infected-cells with isolates of A. baumannii. Each open circle corresponds to the average of two independent experiments in duplicated (n = 4), plotted as the mean ± SD. (A) Bars indicate the TNFα, (C) IL-6 and (E) IL-1β released by A549 infected-cells with each isolate from each hospital comparing blood source vs other sources and (B) the TNFα, (D) IL-6 and (F) IL-1β released by A549 comparing phenotypes of MDR, XDR, PDR or NMDR. NS, non-significant. [file Image_5.tif]

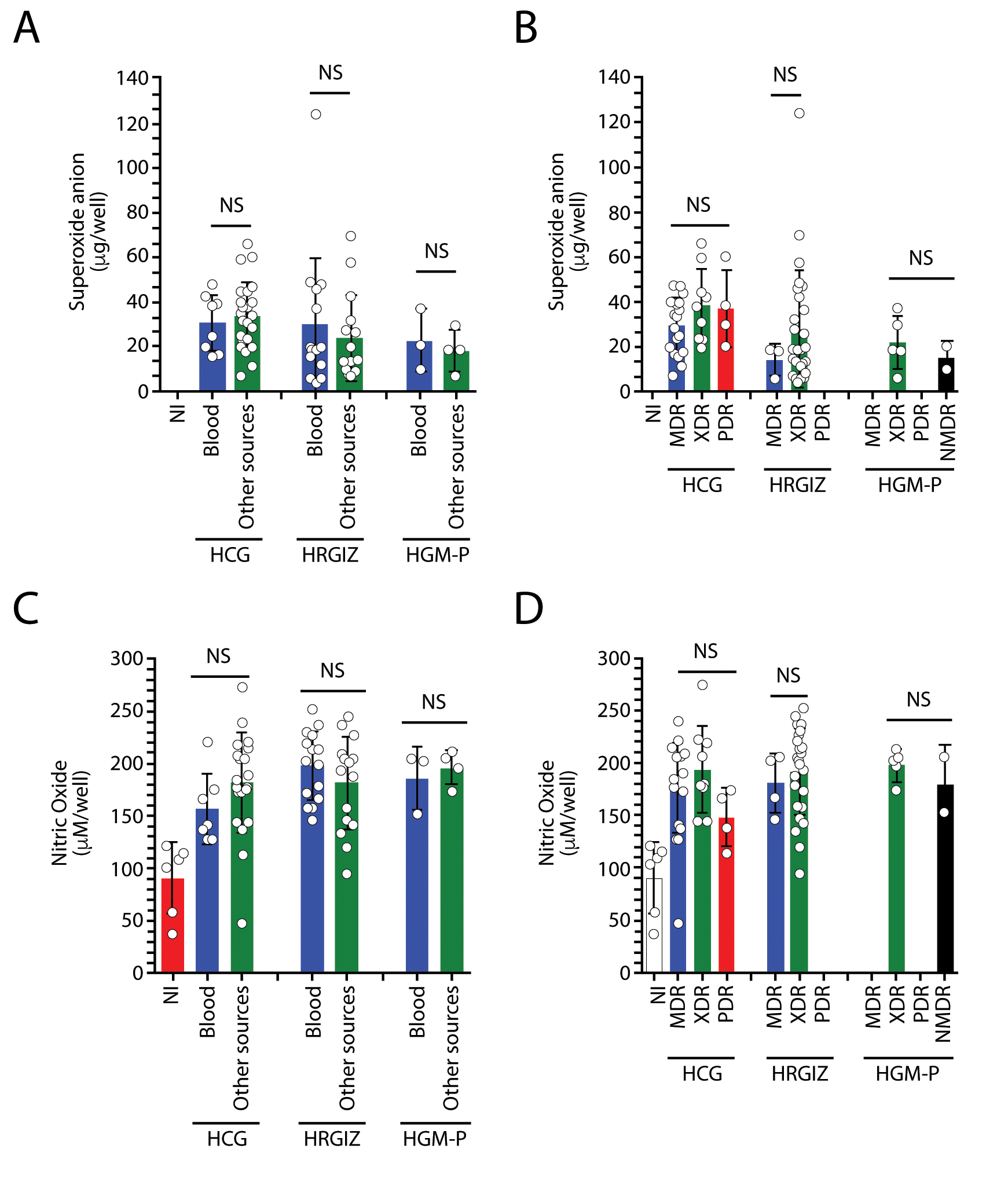

Supplement: FIGURE S6 — Production of oxygen and nitrogen reactive species by A549 infected-cells with isolates of A. baumannii. Each open circle corresponds to the average of two independent experiments in duplicated (n = 4), plotted as the mean ± SD. (A) Superoxide anion or (C) Nitric oxide production by A549 infected-cells with each isolate from each hospital were compared by blood source vs other sources and (B) Superoxide anion or (D) Nitric oxide production by A549 infected-cells by its phenotype of MDR, XDR, PDR or NMDR. NS, non-significant. [file Image_6.TIF]
